# Development of a Multichannel Membrane Reactor with a Solid Oxide Cell Design

**DOI:** 10.3390/membranes13020120

**Published:** 2023-01-17

**Authors:** Hong Huang, Ziyue Guo, Remzi Can Samsun, Stefan Baumann, Nikolaos Margaritis, Wilhelm Albert Meulenberg, Ralf Peters, Detlef Stolten

**Affiliations:** 1 Electrochemical Process Engineering (IEK-14), Forschungszentrum Jülich GmbH, 52425 Jülich, Germany; 2 Material Synthesis and Manufacturing Processes (IEK-1), Forschungszentrum Jülich GmbH, 52425 Jülich, Germany; 3 Engineering and Technology (ZEA-1), Forschungszentrum Jülich GmbH, 52425 Jülich, Germany; 4 Techno-Economic Systems Analysis (IEK-3), Forschungszentrum Jülich GmbH, 52425 Jülich, Germany; 5 Faculty of Mechanical Engineering, RWTH Aachen University, 52072 Aachen, Germany

**Keywords:** membrane reactor, partial oxidation of methane, syngas production, oxygen ion transport membrane, CFD simulation

## Abstract

In this study, we aim to adapt a solid oxide cell (SOC) to a membrane reactor for general chemical reactions to leverage the readily available multichannel design of the SOC. As a proof-of-concept, the developed reactor is tested for syngas production by the partial oxidation of methane using oxygen ion transport membranes (ITMs) to achieve oxygen separation and permeation. A La_0.6_Sr_0.4_Co_0.2_Fe_0.8_O_3−δ_ (LSCF) membrane and Ni/MgAl_2_O_4_ catalyst are used for oxygen permeation and the partial oxidation of methane, respectively. ANSYS Fluent is used to assess the reactor performance with the help of computational fluid dynamics (CFD) simulations. The membrane permeation process is chemical kinetics achieved by user-defined functions (UDFs). The simulation results show that the oxygen permeation rate depends on the temperature, air, and fuel flow rates, as well as the occurrence of reactions, which is consistent with the results reported in the literature. During isothermal operation, the product composition and the species distribution in the reactor change with the methane flow rate. When the molar ratio of fed methane to permeated oxygen is 2.0, the methane conversion and CO selectivity reach a high level, namely 95.8% and 97.2%, respectively, which agrees well with the experimental data reported in the literature. Compared to the isothermal operation, the methane conversion of the adiabatic operation is close to 100%. Still, the CO selectivity only reaches 61.6% due to the hot spot formation of 1491 K in the reactor. To reduce the temperature rise in the adiabatic operation, reducing the methane flow rate is an approach, but the price is that the productivity of syngas is sacrificed as well. In conclusion, the adaption of the SOC to a membrane reactor is achieved, and other reaction applications can be explored in the same way.

## 1. Introduction

Membrane reactors have long been used for chemical reactions that are limited by chemical thermodynamics, kinetics, or both. To date, many membrane reactor concepts have been developed via the coupling of different membranes, including shifts in equilibria, and reactant dosing, as summarized by Morgenstern [1]. A conventional design of a membrane reactor is a cylinder tube. This kind of design is simple but often brings about the problem of concentration polarization, leading to lower productivity. A trend in membrane reactor design is to make the design more compact, thus offering better heat and mass transfer performance and expected higher productivity. For example, Murphy et al. [2] designed a novel ceramic micro-channel reactor for the steam reforming of methane to increase the conversion, yield, and catalyst lifetime and, at the same time to reduce reactor size and weight. The heat efficiency of the reactor is up to 88%, with 100% methane conversion at a space velocity of 15,000 h^−1^. Another similar study of methanol steam reforming in a mesh microchannel reactor was performed by Pan et al. [3]. Different channel cross-sections and distribution patterns were compared, with the finding that the mesh tooth-type channel performs better than the rectangular mesh one in terms of methanol conversion and selectivity. To leverage the advantages of microchannel reactors, Vigneault et al. [4] developed a multi-channel membrane reactor for hydrogen production by the steam reforming of methane. A 91% methane conversion and nearly 100% hydrogen purity were thereby obtained, showing the superiority of combining microchannels and membranes in a reactor.

Motivated by the high performance of the above-mentioned reactors, this study tries to adapt an SOC to a membrane reactor for the first time, which is inspired by its compact multichannel design. To realize this idea, the adapted membrane reactor is applied to the partial oxidation of methane by means of computational fluid dynamics (CFD). In the following sections, the membrane reactor structure will first be demonstrated, after which the procedures for the CFD simulation will be shown in a step-by-step approach. Finally, the performance of the reactor under adiabatic and isothermal conditions will be discussed and compared.

## 2. The Membrane Reactor Concept

**Figure 1:**
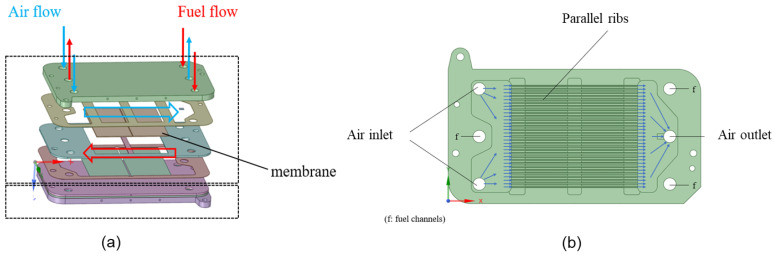
Structure of the membrane reactor prototype (**a**) complete overview; and (**b**) detailed structure and flow of the air domain.

An overview of the membrane reactor prototype is shown in Figure 1. The membrane reactor consists of two layers, each of which has two cells: one is the fuel domain and the other the air domain, as shown in Figure 1a. Figure 1b displays the detailed structure and flow direction of the air domain. The membrane in each layer has two pieces with the same structure, which is for fencing off the air and fuel domains. The fuel domain can be used for various reforming reactions to produce hydrogen, such as the steam reforming of methane or methanol. The membrane can then be a palladium membrane for separating hydrogen. In contrast, the air domain can be used for the sweep gas nitrogen to remove the produced hydrogen. Alternatively, the membrane reactor can be used for the partial oxidation of hydrocarbons, where the fuel domain is used to supply hydrocarbons, and the air domain acts as the oxygen source, with the membrane serving as an ion transport membrane (ITM) for the extraction of oxygen from the air.

## 3. Application to the Partial Oxidation of Methane

In this section, the above membrane reactor will be applied to the partial oxidation of methane.

### 3.1. Reaction Mechanism, Catalyst, and Kinetics

In general, it is believed that the reaction mechanism of the partial oxidation of methane consists of the methane combustion reaction to provide heat to the system and the reforming reactions to generate the desired syngas, as follows:

(1) $$CH_{4}+2O_{2}\to CO_{2}+2H_{2}O\;\Delta H_{298K}=-803\;\mathrm{kJ}/\mathrm{mol}$$

(2) $$CH_{4}+H_{2}O↔CO+3H_{2}\;\Delta H_{298K}=+206\;\mathrm{kJ}/\mathrm{mol}$$

(3) $$CO+H_{2}O↔CO_{2}+H_{2}\;\Delta H_{298K}=-41\;\mathrm{kJ}/\mathrm{mol}$$

(4) $$CH_{4}+2H_{2}O↔CO_{2}+4H_{2}\;\Delta H_{298K}=+165\;\mathrm{kJ}/\mathrm{mol}$$

The most widely used catalysts for the partial oxidation of the methane process are Ni-based catalysts due to their high activity, stability, and strong resistance to carbon formation [5]. Liander [6] first reported the use of Ni-based catalysts and confirmed that Ni containing 15% Al_2_O_3_ is more effective than pure Ni for the formation of CO for the partial oxidation of the methane process. Since then, a large number of studies on Ni/Al_2_O_3_ catalysts have been published [5,7,8]. However, Dissanayake et al. [7] found that the NiAl_2_O_4_ produced from Ni/Al_2_O_3_ deactivated the catalyst, which hinders the methane oxidation process and affects the reforming process for the production of syngas. To solve this problem, Ruckenstein et al. [9] developed several NiO/MgO solid solution catalysts for this reaction. The results exhibit that NiO/MgO solid solution showed better catalytic activity and syngas selectivity than their mechanical mixtures.

Cobalt-based catalysts have also been extensively studied. Similar to Ni-based ones, Co is usually mixed with metal oxides, such as Al_2_O_3_, CaO, and BaO [10,11], for their improvement of the catalytic performance compared to single metal catalysts. But like Ni-based catalysts, the formation of by-products such as CoAl_2_O_4_ and carbon remains a challenge for long-term operation [5]. Among the available catalysts in the literature, Co/MgO is a promising candidate because of its ability to inhibit carbon formation while maintaining high activity in the long run. Still, one drawback is that the operating temperature should be elevated to 1200–1300 °C [12].

In this work, a Ni/MgAl_2_O_4_ catalyst with a density of 2355 kg/m^3^ and porosity of 0.5 [13] was used. The studies of Trimm et al. [14] and Xu and Froment [15] showed that the reaction rates of methane oxidation and steam reforming are related to the adsorption of a gas on the catalyst surface. Corresponding to the reaction mechanism above, the rate equations for reactions Equations (1)–(4) are shown in Equations (5)–(8), respectively [15,16]:

(5) $$\begin{array}{c} r_{1}=\frac{k_{1a}p_{CH_{4}}p_{O_{2}}}{{\left(1+K_{CH_{4},ox}p_{CH_{4}}+K_{O_{2},ox}p_{O_{2}}\right)}^{2}}+\frac{k_{1b}p_{CH_{4}}p_{O_{2}}}{\left(1+K_{CH_{4},ox}p_{CH_{4}}+K_{O_{2},ox}p_{O_{2}}\right)}\;\; \end{array}$$

(6) $$\begin{array}{c} r_{2}=\frac{k_{2}/p_{H_{2}}^{2,5}\left(p_{CH_{4}}p_{H_{2}O}-\frac{p_{H_{2}}^{3}p_{CO}}{K_{eq,2}}\right)}{{\left(1+K_{CO}p_{CO}+K_{H_{2}}p_{H_{2}}+K_{CH_{4}}p_{CH_{4}}+\frac{K_{H_{2}O}p_{H_{2}O}}{p_{H_{2}}}\right)}^{2}}\;\;\; \end{array}$$

(7) $$\begin{array}{c} r_{3}=\frac{k_{3}/p_{H_{2}}\left(p_{CO}p_{H_{2}O}-\frac{p_{H_{2}}p_{CO_{2}}}{K_{eq,3}}\right)}{{\left(1+K_{CO}p_{CO}+K_{H_{2}}p_{H_{2}}+K_{CH_{4}}p_{CH_{4}}+\frac{K_{H_{2}O}p_{H_{2}O}}{p_{H_{2}}}\right)}^{2}} \end{array}$$

(8) $$\begin{array}{c} r_{4}=\frac{k_{4}/p_{H_{2}}^{3,5}\left(p_{CH_{4}}p_{H_{2}O}^{2}-\frac{p_{H_{2}}^{4}p_{CO_{2}}}{K_{eq,4}}\right)}{{\left(1+K_{CO}p_{CO}+K_{H_{2}}p_{H_{2}}+K_{CH_{4}}p_{CH_{4}}+\frac{K_{H_{2}O}p_{H_{2}O}}{p_{H_{2}}}\right)}^{2}} \end{array}$$

where *p*_j_ is the partial pressure of gas component *j* and *K*_eq,i_ is the equilibrium constant of each reversible reaction *i*, which is given in Equations (9)–(11) [13]:

(9) $$\begin{array}{c} K_{eq,2}=\exp\left(-\frac{26,830}{T}+30.144\right)\;bar^{2}\;\; \end{array}$$

(10) $$\begin{array}{c} K_{eq,3}=\exp\left(\frac{4,400}{T}-4.036\right) \end{array}$$

(11) $$\begin{array}{c} K_{eq,4}=K_{eq,2}K_{eq,3}=\exp\left(-\frac{22,430}{T}+26.078\right)\;bar^{2}\; \end{array}$$

The Arrhenius reaction rate *k_i_* and catalyst adsorption equilibrium constant *K*_j_ of Equations (5)–(8) can be expressed as Equations (12)–(13):

(12) $$\begin{array}{c} k_{i}=A_{i}\exp\left(-\frac{E_{act,i}}{RT}\right)\;\;\; \end{array}$$

(13) $$\begin{array}{c} K_{j}=A(K_{j})\exp\left(-\frac{\Delta H_{j}^{0}}{RT}\right)\;\;\; \end{array}$$

where the Arrhenius parameters $A_{i}$, $E_{act,i}$, *A(K_j_)* and $\Delta H_{j}^{0}$ were provided by Smet et al. [16] and Xu and Froment [15], as shown in Table 1 and Table 2.

### 3.2. Oxygen Permeation Membrane

The oxygen ITMs are ceramic membranes that are highly permeable to oxygen. Compared to the industrial production of oxygen via cryogenic distillation, the use of ITMs to provide oxygen requires less energy and is, therefore, more efficient. With the advancement of technology in recent decades, many ITMs have been developed and tested. Deibert et al. [17] comprehensively reviewed the development progress of ion-conducting ceramic membrane reactors. Table 3 displays representative oxygen fluxes and the operating conditions of various ITMs from the literature [18,19,20,21,22,23].

From the table, the oxygen permeability of different membranes varies significantly, among which BSCF (Ba_0.5_Sr_0.5_Co_0.8_Fe_0.2_O_3−δ_), LSCF (La_0.6_Sr_0.4_Co_0.2_Fe_0.8_O_3−δ_) and SCFN (SrCoxFe_0.9-x_Nb_0.1_O_3−δ_) show better performance. In addition to the material type, parameters such as temperature, feed gas flow rate, sweep gas flow rate, oxygen partial pressure, the distance between the membrane and gas inlet, and membrane thickness can also affect the permeability of a membrane, which the working temperature is a key factor affecting oxygen permeability. In particular, LSCF-type membranes are much more sensitive to temperature changes. Compared to other membrane types, LSCFs are more suitable for applications at higher temperatures, from 750 to 950 °C [24]. BSCF-type membranes also have high oxygen permeability at lower temperatures, but BSCF membranes have poor structural stability in a hydrogen-containing environment [18]. In contrast, LSGF-BSCF-type membranes can maintain their structure in a hydrogen-containing environment at 900 °C, although their oxygen permeability is significantly lower throughout the same temperature regime [18]. SCFN is a relatively new material; this membrane type exhibits a fairly high oxygen permeability but can only work well in a non-reactive environment where carbon dioxide is on the permeate side. It remains a long way from use in practical applications [22,25,26]. For SCF and LNO membranes, even at higher temperatures, only lower oxygen permeation rates could be measured [20,21,23]. In view of the better oxygen permeability and larger working temperature range, only the LSCF membranes will be more specifically introduced and used in the later simulations.

The numerical model of the oxygen permeation of the LSCF membrane has been thoroughly studied. Xu et al. [24] provided a revised form of the equation that describes the oxygen permeability of LSCF membranes, as shown in Equation (14):

(14) $$\begin{array}{c} {\;J}_{O_{2\;}}=\frac{D_{V}k_{r}\left(P_{O_{2}}^{\prime 0.5}-P_{O_{2}}^{\prime \prime 0.5}\right)}{2{Lk}_{f}{\left(P_{O_{2}}^{\prime }P_{O_{2}}^{\prime \prime }\right)}^{0.5}+D_{V}\left(P_{O_{2}}^{\prime 0.5}+P_{O_{2}}^{\prime \prime 0.5}\right)}\; \end{array}\;$$

The activation energy and pre-exponential coefficients of the temperature-relevant parameters $D_{V}$ (oxygen vacancies diffusion coefficient), $k_{r}$ and $k_{f}$ (surface exchange rate constants) were obtained through experiments. The main variables in this equation are temperature, membrane thickness L, and oxygen partial pressure $P_{O_{2}}$ on both membrane sides. In addition, the oxygen permeability at 750–950 °C and different feed side oxygen partial pressures $P_{O_{2}}^{\prime }$ (0.1–1 atm) were measured. It was concluded that oxygen permeation depends on the recombination rate of oxygen ions at low temperatures and is dominated by bulk diffusion at high temperatures [24]. Later, Tan et al. [27] confirmed the assumption made by Xu et al. [24] that bulk diffusion and surface exchange kinetics only depend on temperature. Hong et al. [19] studied the influence of gas flow, transport, and reaction on oxygen permeability. This study also considered the local oxygen concentration on the membrane surface, so the activation energy and pre-exponential coefficients of temperature-relevant parameters $D_{V}$, $k_{r}$ and $k_{f}$ in Xu’s model were modified, as shown in Equations (15)–(17):

(15) $$\begin{array}{c} \begin{array}{c} D_{V}=D_{V}^{0}\exp\left(-\frac{E_{D}}{RT}\right)\; \end{array} \end{array}\;$$

(16) $$\begin{array}{c} k_{r}=k_{r}^{0}\exp\left(-\frac{E_{r}}{RT}\right)\; \end{array}\;$$

(17) $$\begin{array}{c} k_{f}=k_{f}^{0}\exp\left(-\frac{E_{f}}{RT}\right)\;\;\; \end{array}\;$$

where *R* is the universal gas constant, *T* is the temperature of the membrane surface, and the pre-exponential factor $k_{f}^{0}$, $k_{r}^{0}$, $D_{V}^{0}$ and the activation energy $E_{f}$, $E_{r}$ and $E_{D}$ are derived from experimental data [19,24], which are placed in Table 4, provided by Hong et al. [19]. It should be noted that the oxygen partial pressure $P_{O_{2}}^{\prime }$ and $P_{O_{2}}^{\prime \prime }$ in Equation (14) represent local values (near the membrane surface).

### 3.3. CFD Simulation

This sub-section introduces the procedures of CFD simulation for the developed membrane reactor.

#### 3.3.1. Geometry and Modification

**Figure 2:**
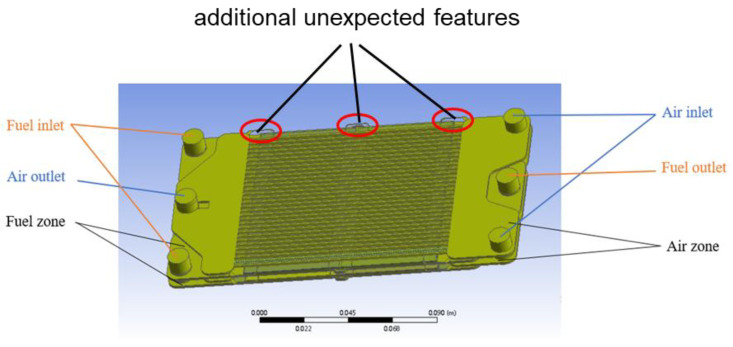
The generated geometry of the fluid domain via filling.

In order to perform the CFD simulations, the geometry of the fluid domain must first be generated. Here, the filling method is used to generate the fluid domain. All of the inlets and outlets are blocked to create a closed space for filling the entire geometry, and the fluid domains of the air and fuel zones are obtained separately. The generated geometry of the fluid domain is shown in Figure 2.

However, it can be seen from the above figure that there are some defects in the fluid domain. As the components of the reactor are not completely matched with each other during assembly, many small, unwanted features are caused in the generated fluid domain, in particular, the features near the ribs in the air zone, as shown by the red marks in Figure 2. Due to the irregular shape, these defects make the simulations unsteady and hard to converge.

A better way to resolve this problem is to reconstruct the model by retaining the dimension parameters such as the reactor inlet and outlet surface diameters, as well as the characteristics of the planar ribs in the air zone, and the thickness and area of the oxygen permeable membranes the same as the original model.

**Figure 3:**
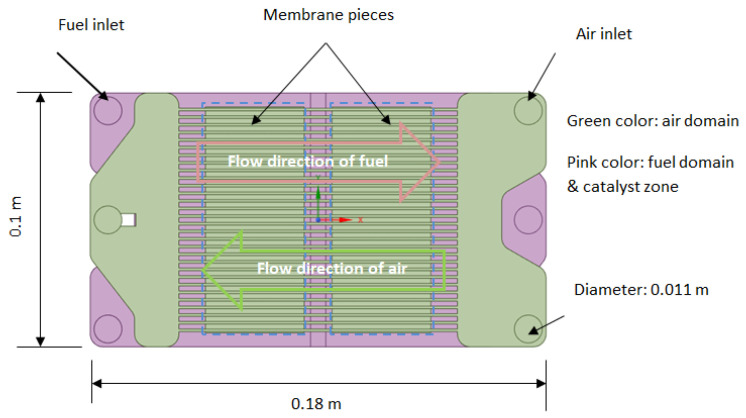
The reconstructed total fluid domain.

The reconstructed total fluid domain is shown in Figure 3. The part with the green color represents the air domain, which consists of two parallel structures that communicate with each other through three connecting pipes; two for the air inlet and one for the outlet. The middle section of the airflow zone is composed of 31 ribs, each with a width of 1 mm and the distance of the two neighboring ribs being 2 mm. Other dimensions are shown in Table 5. The part with pink color represents the fuel domain, which is also the catalyst zone. The partition used to seal the gap between the two membranes results in a groove, which is represented in the modified geometry. The Ni-mesh placed in the reactor is merged with the fuel domain due to its small scale for the same reason of facilitating simulations.

#### 3.3.2. Model Settings

Two cases under isothermal and adiabatic conditions will be simulated using ANSYS Fluent version 2020 R2 as CFD software. The realizable k-epsilon turbulence model is selected. The non-equilibrium wall function is recommended for simulating fluid flows involving separation, reattachment, and impingement, according to the literature [28]. Considering the separation of oxygen near the membrane surface and its formation on the other side of the membrane during the permeation process, the non-equilibrium wall function is selected for the near-wall treatment. The energy equation was turned on due to the need for energy conversion to account for the heat effect of the chemical reactions. Various species and reactions were involved, described through the species transport model with the finite-rate volumetric reaction enabled. Moreover, diffusion energy source, full multicomponent diffusion, and thermal diffusion of species are checked for the species transport model. It should be noted that the gas composition in user-defined functions (UDFs) must be consistent with the order in which it was set in the mixture. The standard scheme is applied to the pressure. In contrast, the second-order upwind schemes are selected for other parameters such as density, momentum, turbulence kinetic energy, turbulence dissipation rate, species, and energy for spatial discretization.

#### 3.3.3. Boundary and Cell Zone Conditions

The settings for the boundary and cell zone conditions in this work are listed in Table 6. Various feed velocities and temperatures are considered for the sensitivity analyses. The mass fraction of the air and fuel feed flow was based on the composition of air and pure methane. The temperature of the wall of the fuel zone and membrane surface is a fixed value for the isothermal operation. For the adiabatic operation, the heat transfer on both sides of the contact surface between the membranes and fuel zone was set to be coupled, and the outside wall had no heat flux. The source term option must be enabled for both the air and fuel zones in order to realize the oxygen permeation, which will be discussed in the next section, and the reaction option must be enabled in order for the fuel zone to simulate the chemical reactions.

As mentioned earlier, the oxygen permeation process and chemical reaction kinetics are implemented by UDFs. The DEFINE_ADJUST is a general-purpose macro that can be used for modifying variables that are not passed as arguments in a function. Here, it is used for locating the position of the membrane wall and the adjacent cells on both of its sides. After this, the oxygen flux is calculated and stored in these adjacent cells via user-defined memories. Then, the DEFINE_SOURCE macro reads these stored flux values and incorporates them into the continuity and energy equations, and the membrane permeation is realized in this way. The DEFINE_VR_RATE is a dedicated macro for customizing volumetric reaction kinetics and is not in the standard form. By passing the arguments of temperature, mass fraction, and pressure, the function returns the reaction rates.

### 3.4. Results and Discussion

The CFD simulation results of the developed models will be presented and discussed in this section. The membrane permeation performance without reaction will first be tested for validation. The advantages and disadvantages of the isothermal and adiabatic operations will then be compared.

#### 3.4.1. Geometry and Modification

According to Equation (14), the oxygen permeability is determined by temperature T, membrane thickness L and oxygen pressure on both sides of the membrane $P_{O_{2}}^{\prime }$ and $P_{O_{2}}^{\prime \prime }$, as well as external factors such as flow rates of air and fuel. In order to separately demonstrate the permeation process, the dependence of the oxygen permeation rate on the above parameters will be assessed without adding chemical reactions in the fuel zone. Afterward, chemical reactions will be enabled at various methane flow rates to clarify the influence of the reaction on the permeation rate of oxygen, which will be compared with the case without a reaction. The results are shown below.

**Figure 4:**
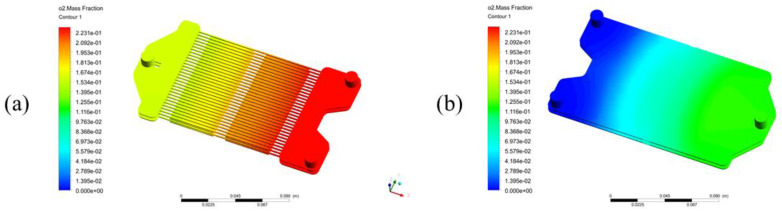
The distribution of the oxygen mass fraction under the above-described conditions in (**a**) the air zone and membranes, the flow direction is from right to left; and (**b**) the fuel zone, the flow direction is from left to right.

At 1100 K and atmospheric pressure, with an airflow rate of $1.314\times 10^{-6}\;\mathrm{kg}/s$ and fuel flow rate of $8.772\times 10^{-7}\;\mathrm{kg}/s$, the distribution of the oxygen mass fraction in the reactor is shown in Figure 4. The changes in oxygen concentration only take place on each side of the membranes. The oxygen mass fraction in the air zone (Figure 4a) continuously decreases along the membrane surfaces in the stream’s direction. But in the fuel zone (Figure 4b), along the membrane surfaces in the stream direction, the oxygen fraction continuously increases.

**Figure 5:**
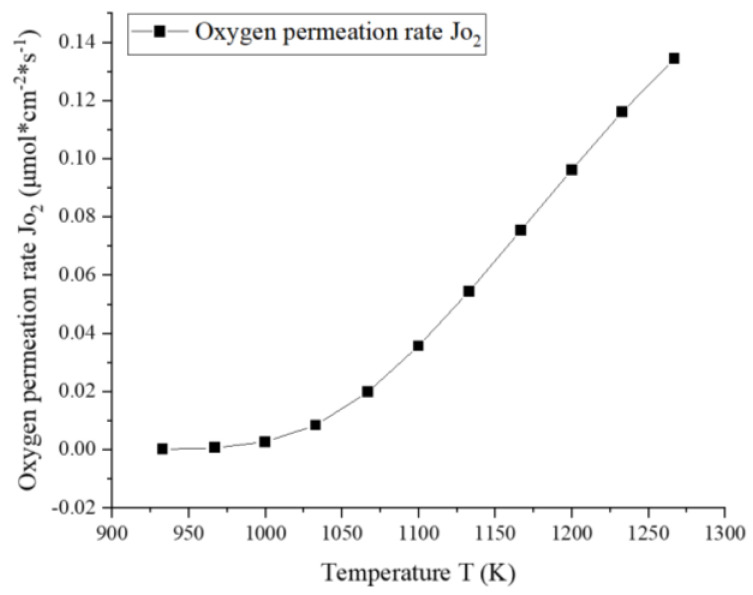
The dependence of oxygen permeation rate on temperature ($m_{air}=1.971\times 10^{-5}\;\mathrm{kg}/s$, $m_{fuel}=1.462\times 10^{-6}\;\mathrm{kg}/s$).

The dependence of the oxygen permeation rate on the temperature is shown in Figure 5. It should be noted that the plotted values in the figure are the surface-averaged oxygen permeation rates. The simulations are performed with a sufficiently high airflow rate so that enough oxygen can be supplied over the entire temperature range. The simulation results suggest that the relationship between the permeation flux and temperature follows a power-law form. The flux increases slowly at temperatures below 1100 K, above which the flux increases much faster. This is mainly determined by the surface exchange rates $k_{f}$ and $k_{r}$, and the oxygen vacancy diffusion coefficient $D_{V}$ in Equation (14), which are strongly related to temperature. The oxygen permeation fluxes obtained at different temperatures are within a reasonable range compared with the reported experimental data on LSCF membranes [24,27,29,30].

**Figure 6:**
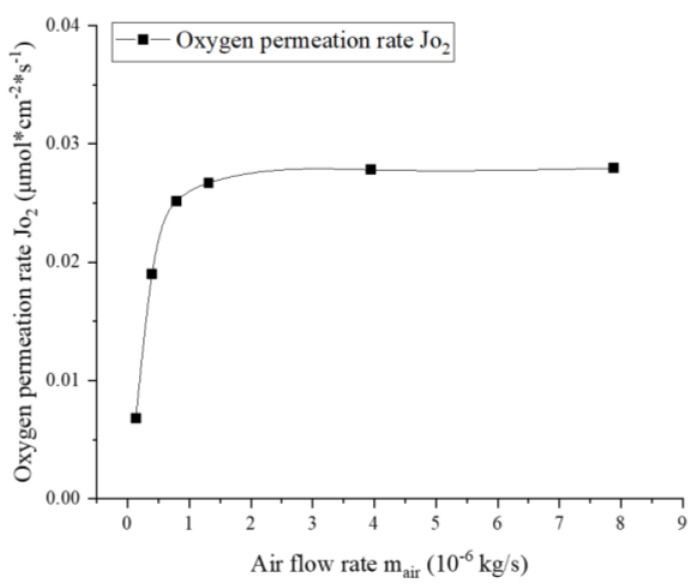
Dependence of the oxygen permeation rate on airflow rate ($m_{fuel}=8.772\times 10^{-7}\;\mathrm{kg}/s$, T=1100 K).

Next, the dependence of the oxygen permeation rate on the airflow is studied and shown in Figure 6. The simulations were carried out at 1100 K with a constant fuel flow rate of $8.772\times 10^{-7}\;\mathrm{kg}/s$. The permeation flux increases very quickly at low airflow rates and then reaches a maximum value when the airflow rate is higher than $1.5\times 10^{-6}\;\mathrm{kg}/s$. This characteristic shows good agreement with the experimental results reported by Luo et al. [31]. This is because, under extremely low airflow rates, the concentration and partial pressure of oxygen in the air zone drop rapidly during permeation, which is the limiting factor to the flux. At this time, increasing the feed airflow rate is effective in boosting the oxygen pressure $P_{O_{2}}^{\prime }$ in the air zone, thereby rapidly increasing the permeability. At higher airflow rates, however, due to sufficient oxygen content in the air, the limiting factor is transferred to the membrane itself. At this point, further increasing the amount of air has little effect on the permeation flux.

**Figure 7:**
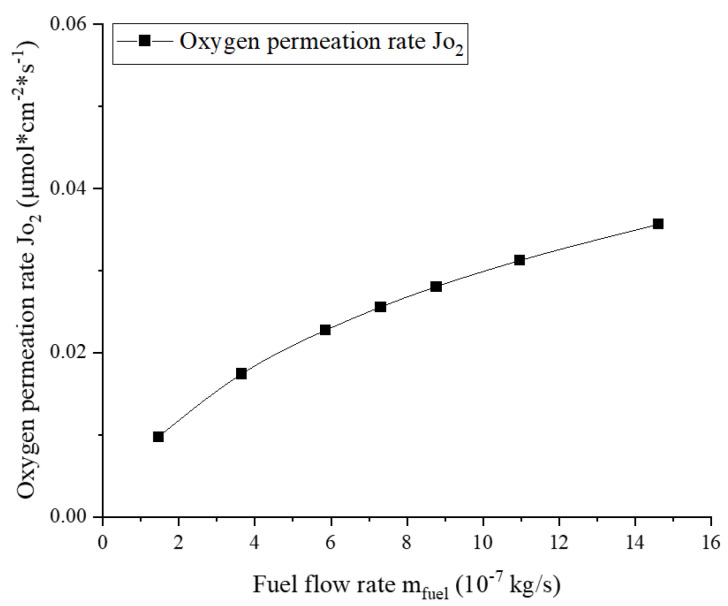
Dependence of the oxygen permeation rate on the fuel flow rate ($m_{air}=1.971\times 10^{-5}\;\mathrm{kg}/s$, T=1100 K).

Similarly, the dependence of the oxygen permeation rate on the fuel flow is displayed in Figure 7. The simulations were carried out at 1100 K with a relatively higher airflow rate of $1.971\times 10^{-5}\;\mathrm{kg}/s$. The simulation results show that the oxygen permeation rate increases with the fuel flow rate. This is because the increased fuel stream will reduce the oxygen concentration on the permeate side and thereby reduce the oxygen pressure $P_{O_{2}}^{\prime \prime }$. This characteristic has also been confirmed by Hong et al. [19] and Habib et al. [32].

#### 3.4.2. Effect of Reactions on Oxygen Permeation

**Figure 8:**
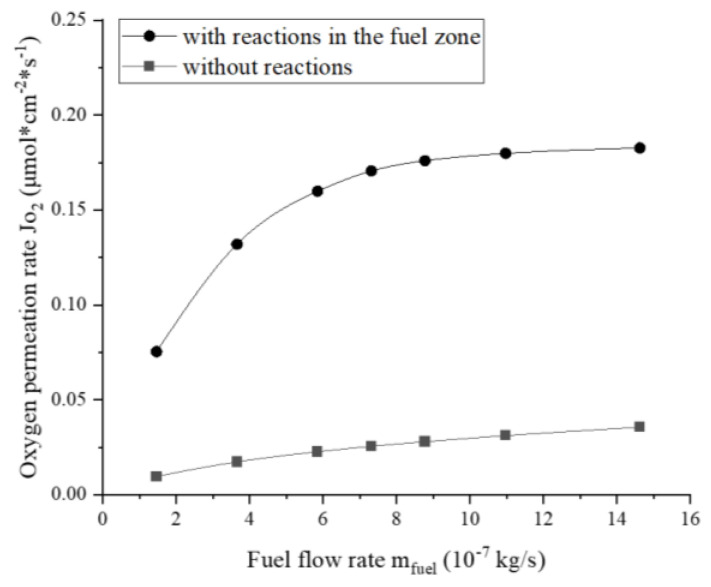
Effect of the reactions on the oxygen permeation rate at different fuel flow rates ($m_{air}=1.971\times 10^{-5}\;\mathrm{kg}/s$, T=1100 K).

In addition to increasing the flow rate of fuel, the oxygen pressure on the permeate side $P_{O_{2}}^{\prime \prime }$ can be further reduced by enabling the reactions in the simulation. Figure 8 compares the oxygen permeation fluxes with and without reactions with respect to the fuel flow rate. The simulation results show that when chemical reactions exist in the fuel zone, the oxygen permeation rate increases by 4.1–6.7 times, depending on the fuel flow rates. At a lower fuel flow rate, the oxygen permeation rate increases faster with the fuel flow rate. This is because insufficient methane feed slows the oxygen consumption rate. Xu et al. [24] found that adding methane to the permeate side of LSCF membranes during the experiment raised the oxygen permeation rate by more than five times, which agrees well with the results of this simulation. In addition, the study by Hong et al. [19] showed that the chemical reaction on the permeate side could increase the oxygen permeation rate by 2–9 times. Therefore, the simulation results are deemed reasonable.

#### 3.4.3. Isothermal Operation

As previously noted, the oxygen permeation rate varies strongly with temperature. A moderate temperature of 1100 K was selected as the operating temperature of the reactor, which is also a reasonable value in the literature from 973–1173 K [9,33,34]. In the following content, the properties arising from the isothermal operation of the reactor under different methane flow rates will be presented, including the product composition, methane conversion, and CO selectivity, as well as average volumetric reaction rates and species distribution in the reactor.

The stoichiometric ratio of fuel to oxygen has a major impact on the reactions, and this is also true in membrane reactors. However, as previously noted, the oxygen permeation flux also changes with the fuel flow rate, so the stoichiometric ratio of fuel to oxygen is not completely linear with respect to the amount of fuel. Therefore, the relationship between the fed methane to the permeated oxygen molar ratio and methane flow rate must first be analyzed.

**Figure 9:**
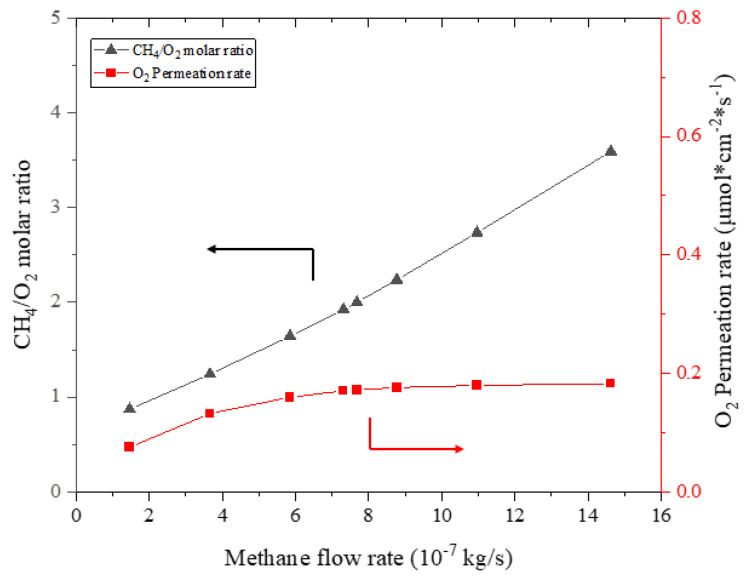
Fed methane to permeated oxygen molar ratio (left y-axis) and oxygen permeation rate (right y-axis) under different methane flow rates ($m_{air}=1.971\times 10^{-5}\;\mathrm{kg}/s$, T=1100 K).

Figure 9 shows the fed methane to permeated oxygen molar ratio under different methane flow rates in the reactor. The dependence of the oxygen permeation rate on the methane flow rate is also expressed. The oxygen permeation rate increases with the methane flow rate, which is consistent with the analysis presented in the previous section. But the increased rate of permeated oxygen is very moderate, especially at higher methane flow rates, compared to the increase in the methane stream. Therefore, increasing the methane flow rate will still lead to an almost linear increase in the fed methane to the permeated oxygen molar ratio.

**Figure 10:**
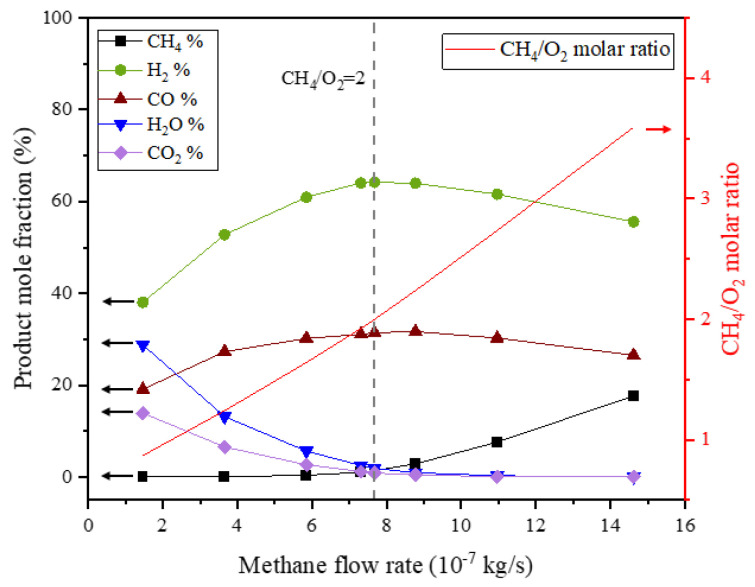
Product composition under different methane flow rates ($m_{air}=1.971\times 10^{-5}\;\mathrm{kg}/s$, T=1100 K). The arrows in the figure indicate the correspondence of the curves and the axes.

The changes in the methane flow rate lead to different product compositions in the reactor outlet, as shown in Figure 10. In all simulation cases, the oxygen was almost completely consumed, so it is not present in the product composition. The simulation results show that when the methane flow rate is relatively low, and the molar ratio of fed methane to permeated oxygen is less than 2.0, the increased methane flow rate leads to increases in CO and H_2_ in the product composition and consequently to decreases in CO_2_ and H_2_O, as the increased methane stream promotes reforming reactions. When the methane flow rate is relatively high, and the molar ratio of fed methane to permeated oxygen is greater than 2.0, the concentration of CO_2_ and H_2_O in the product further decreases with the increased methane stream. The increased methane flow rate simultaneously leads to a decrease in CO and H_2_ and an increase in methane in the product composition resulting from the dilution of the excess methane.

Combining the simulation results with the reaction equations, the product composition can be predicted based on the stoichiometric ratio of methane to oxygen. The prediction shows that when methane is excessive, partial oxidation will mainly occur in the reactor in the generation of CO and H_2_, and excessive methane will also exist in the product. When methane is insufficient, methane combustion will occur. At this time, the main products are CO_2_, H_2_O, and excess oxygen. When the methane-to-oxygen ratio is between 0.5 and 2.0, both reactions will dominate, and the product will be mainly composed of CO, H_2_, CO_2_, and H_2_O. Since this reactor is developed for the production of syngas, it is worth discussing when the methane-to-oxygen ratio is 0.5–2.0 or greater than 2.0, which is also the focus of this section.

**Figure 11:**
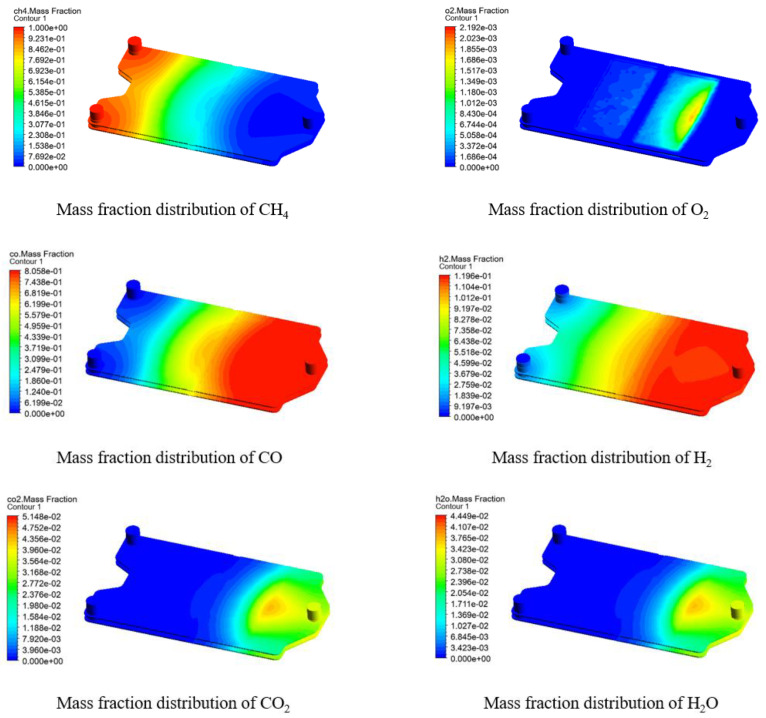
Distribution of species in the fuel zone of the reactor at CH_4_/O_2_ = 2.0 ($m_{air}=1.971\times 10^{-5}\;\mathrm{kg}/s$, $m_{fuel}=7.676\times 10^{-7}\;\mathrm{kg}/s$, T=1100 K).

The species distribution in the fuel zone is more vividly visualized by the contours in Figure 11, which is obtained at a molar ratio of fed methane to permeated oxygen of 2.0. Because of a relatively sufficient methane feed, its mass fraction is not reduced to a low level until it reaches the middle of the fuel zone, during which the permeated oxygen is immediately consumed. After that, the consumption rate of the permeated oxygen slowed down, but the reverse process of the reforming reactions also generated methane, so the oxygen was also completely consumed. During the whole process, the mass fractions of H_2_ and CO both increase almost continuously due to the high methane feed. CO_2_ and H_2_O show very similar trends, with both being produced in small amounts at the membrane surface close to the outlet, as the complete oxidation of methane is predominant at this location.

Methane conversion and CO selectivity are two important parameters for evaluating the performance of the membrane reactors, which are defined as follows:

(18) $$X_{CH4}=\frac{n_{in,CH4}-n_{out,CH4}}{n_{in,CH4}}$$

(19) $$S_{CO}=\frac{1}{2}\cdot \frac{n_{CO}}{n_{in,CH4}-n_{out,CH4}}$$

where $n_{in,CH4}$ and $n_{out,CH4}$ represent the methane molar flow at the reactor inlet and outlet. $n_{CO}$ is the production rate of carbon monoxide at the reactor outlet.

**Figure 12:**
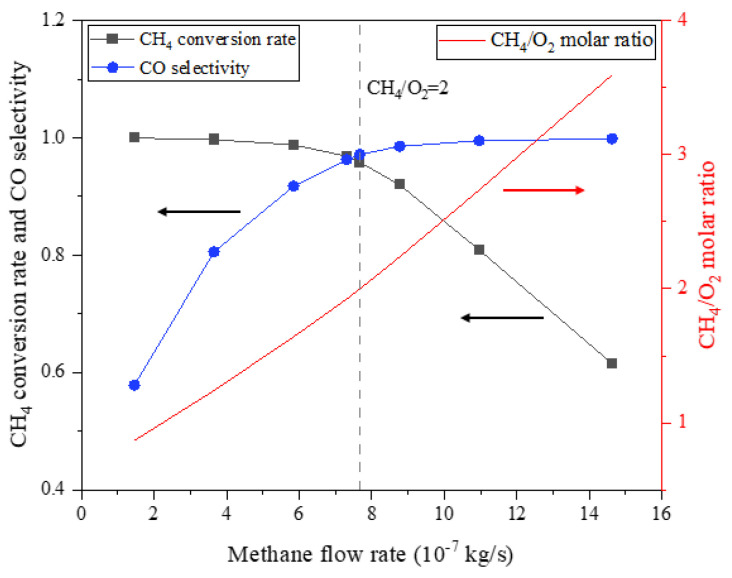
Methane conversion rate and CO selectivity under different methane flow rates ($m_{air}=1.971\times 10^{-5}\;\mathrm{kg}/s$, T=1100 K). The arrows in the figure indicate the correspondence of the curves and the axes.

Figure 12 displays the methane conversion and CO selectivity in the above simulation, of which the molar ratio of fed methane to permeated oxygen is simultaneously expressed as a reference. The simulation results indicate that the increased methane stream continuously leads to a decrease in the methane conversion rate and an increase in CO selectivity, which is due to the increasing dominance of the reforming reactions. In particular, when the molar ratio of fed methane to permeated oxygen corresponded to the reactants’ stoichiometric ratio, namely 2.0, both the methane conversion rate and CO selectivity achieved relatively high levels, of 95.8% and 97.2%, respectively. According to Jin et al. [35], who conducted an experiment on the partial oxidation of methane using a membrane reactor with the same LSCF membrane and Ni/Al_2_O_3_ catalyst, the methane conversion rate and CO selectivity achieved more than 96% and over 97%, respectively, at temperatures between 1098 K and 1158 K and under a methane-to-oxygen ratio of around 2.0, which is highly consistent with the simulation results in this work, and so the results are considered reliable.

#### 3.4.4. Adiabatic Operation

Because of the limitation of membrane materials and the requirements of the reactions, the temperature distribution in the reactor is an important parameter in adiabatic operation. This section will simulate selected cases of adiabatic operation and then propose strategies for controlling the temperature.

**Figure 13:**
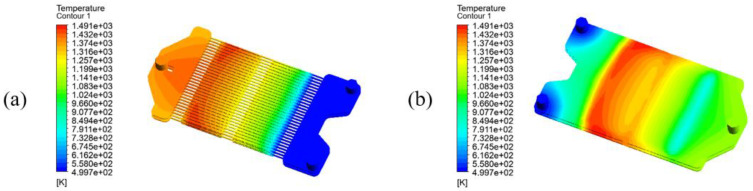
Temperature distribution under adiabatic operation in (**a**) the air zone and membranes; (**b**) the fuel zone. ($m_{air}=1.164\times 10^{-5}\;\mathrm{kg}/s$, $m_{fuel}=4.532\times 10^{-7}\;\mathrm{kg}/s$, $T_{air,\;in}=T_{fuel,\;in}=500\;K$).

The temperature distribution of the reactor with both air and methane inlet temperature of 500 K under adiabatic conditions with an airflow rate of $1.164\times 10^{-5}\;\mathrm{kg}/s$ and a fuel flow rate of $4.532\times 10^{-7}\;\mathrm{kg}/s$ is shown in Figure 13.

It can be seen that under these operating conditions, the maximum temperature in the reactor reaches 1491 K, which exceeds the maximum allowable membrane temperature of 1273 K [19]. During further simulations, it was found that increasing the airflow rate or decreasing the methane stream can lead to a decrease in both reactor’s maximum temperature and the fuel zone outlet temperature. In other words, although the maximum temperature in the reactor can be lowered to a temperature range suitable for the membrane, the reaction temperature will also be lowered at the same time, which will result in relatively low reaction rates. Therefore, the difference between the maximum temperature and outlet temperature in the fuel zone is expected to be reduced. At the same time, it is found that the lower fuel outlet temperature is due to the huge temperature difference between the fuel and air zones on both sides of the membrane, and the air heating takes away a lot of the reaction heat from the fuel zone. Therefore, the air feed temperature must be increased to reduce the temperature difference in the reactor, and the airflow rate must also be increased so that the temperature rise will be reduced, which helps reduce the maximum temperature in the reactor.

**Figure 14:**
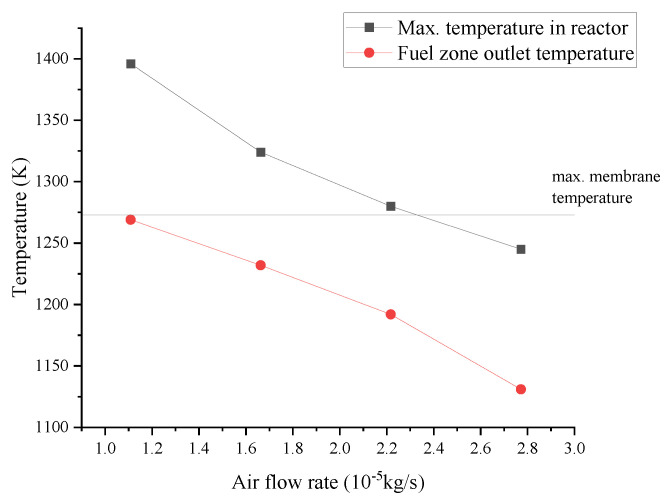
The maximum and outlet temperature in the fuel zone under different airflow rates ($m_{fuel}=1.850\times 10^{-7}\;\mathrm{kg}/s$, $T_{air,\;in}=T_{fuel,\;in}=1050\;K$).

Based on the above results, the temperature of the feed air and fuel is adjusted to 1050 K, and the airflow rate is increased to control the temperature in the reactor. Figure 14 shows the maximum and outlet temperature in the fuel zone with both the air and methane feed temperature of 1050 K under different airflow rates. It can be seen that when the feed temperature is 1050 K, the temperature difference in the fuel zone is significantly smaller compared to that at 500 K. In addition, the maximum and outlet temperature of the fuel zone decreases with the increasing air stream because the increased air quantity has improved the heat capacity of the heat removal and better cooling. However, it must be noted that the maximum allowable membrane temperature (1273 K) is taken as a reference for this analysis. In practice, the original reactor for SOC can withstand a maximum of 1100 K, which allows only isothermal operation according to the results of our simulations. In order to realize the adiabatic operation with the existing reactor geometry, either the reactor materials have to be adapted to higher temperatures for partial oxidation operation, or the feed inlet temperatures have to be reduced at the expense of poorer reactor performance.

**Figure 15:**
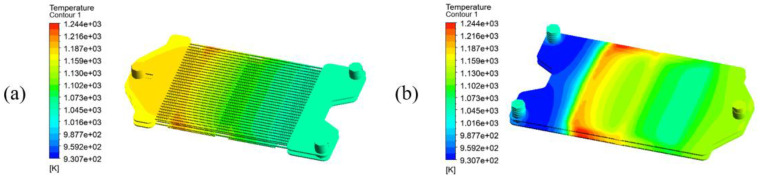
Temperature distribution under adiabatic operation in (**a**) the air zone and membranes; and (**b**) the fuel zone. ($m_{air}=2.772\times 10^{-5}\;\mathrm{kg}/s$, $m_{fuel}=1.850\times 10^{-7}\;\mathrm{kg}/s$, $T_{air,\;in}=T_{fuel,\;in}=1050\;K$).

When the airflow rate reaches $2.772\times 10^{-5}\;\mathrm{kg}/s$, the maximum temperature in the fuel zone is within the allowable temperature range of the membrane, and so the operation of the reactor under this condition is feasible. Overall, the temperature of the air zone in Figure 15a gradually increases along the airflow direction in the reacting area, which is caused by the heating of the fuel zone. In Figure 15b, it can be seen that the temperature of the fuel zone continues to decrease before it arrives at the membrane location as a result of the reforming reactions. When the fuel stream just contacts the membrane surface, due to the permeated oxygen, the complete oxidation of methane is dominant. A large amount of heat is released, and therefore the maximum temperature of 1245 K in the reactor is obtained at that location. Afterward, due to more reforming reactions in the fuel zone and air cooling in the air zone, the temperature decreases continuously. At the outlet of the fuel zone, the product temperature is stable at 1131 K. It should be noted that due to the relatively high temperature of the feed air, the oxygen permeation rate is high at all membrane locations, which leads to a high degree of utilization of the membrane.

Yet, compared with the isothermal operation, the share of complete oxidation of methane in the reactor increased due to high local temperature, resulting in decreases of CO and H_2_ and increases of the CO_2_ and H_2_O fraction in the product. Table 7 presents the product composition, methane conversion rate, and CO selectivity under the above operating conditions. It can be seen that in addition to syngas, there was a large amount of CO_2_ and H_2_O in the product. Although methane was almost completely converted, the CO selectivity only reached around 62%. Therefore, the performance of the reactor during adiabatic operation is worse than that during isothermal operation.

However, the adiabatic simulation provided the operational possibility of the reactor without external temperature control. Assuming that an external heat exchanger is applied, it is theoretically feasible to heat the inlet airflow from room temperature to 1050 K using the heat of the outlet air stream, and the heat of reaction product can also be used for fuel preheating, and therefore the reactor can potentially be operated without external energy input.

Table 8 compares the performance of the reactor in isothermal and adiabatic operations. The methane flow rate during adiabatic operation is significantly lower than during isothermal operation in order to avoid runaway temperatures. A lower methane flow rate and moderate temperature result in a lower oxygen permeation rate during adiabatic operation. Since an increased share of complete oxidation of methane takes place in the fuel zone during adiabatic operation, the CO selectivity is significantly lower than during isothermal operation. Although the performance of the adiabatic operation is poorer, it imposes no requirements on the external temperature control, and the conditions are easier to achieve during actual operation.

## 4. Conclusions

In this study, an SOC was adapted to a multichannel membrane reactor and applied to the partial oxidation of methane for the first time. A CFD model of a membrane reactor combining oxygen permeation and the partial oxidation of methane to produce syngas was developed and simulated under isothermal and adiabatic conditions. The membrane permeation mechanism was based on surface exchange and intra-membrane diffusion applied to the oxygen permeation of LSCF membranes in the reactor. The partial oxidation of methane kinetics, combining complete oxidation and reforming reactions, was applied to the Ni/MgAl_2_O_4_ catalyst in the reactor. During the simulations, oxygen permeation and chemical reactions were implemented by means of user-defined functions.

The oxygen permeability is dependent on the operating temperature, air, and fuel flow rates, as well as the occurrence of chemical reactions. Among these, the temperature is the most influential factor, and so the increased temperature promoted oxygen permeation. When the airflow rate was extremely low, the oxygen permeability mainly depended on the air stream, but it had little effect on the permeability at relatively high airflow rates. The increased fuel flow resulted in a continuous increase in oxygen permeability. The presence of chemical reactions in the fuel zone increased the oxygen permeability by 4.1–6.7 times. The above-mentioned oxygen permeation characteristics are highly consistent with those reported in the literature.

When the reactor was operated at an isothermal temperature of 1100 K, different methane inlet flow rates resulted in different fed methane to permeated oxygen molar ratios, thereby leading to different product compositions. The higher methane flow rate promoted the reforming reactions, and so more products such as CO and H_2_ were generated. The increased molar ratio of fed methane to permeated oxygen resulted in decreased methane conversion and increased CO selectivity. However, at a methane-to-oxygen ratio of 2.0, both the methane conversion rate and CO selectivity achieved high levels, namely 95.8% and 97.2%, respectively, which are close to the experimental data reported in the literature.

Operation of the reactor under adiabatic conditions is theoretically possible but must be carried out at a relatively high feed temperature and air stream and with a low methane stream in order to control the maximum temperature and keep the temperature difference in the reactor within an acceptable range. According to the simulation results, the methane conversion rate and CO selectivity under adiabatic conditions were close to 100% and 61.6%, respectively. Compared to the isothermal operation, the performance was poorer, as the heating of air and the fuel flow requires a lot of reaction heat. The complete oxidation of methane was more dominant than under isothermal conditions, which reduced the generation capability of syngas. However, the adiabatic operation requires no temperature control and no external energy input, so it is of great significance for future applications.

In conclusion, the adaption of an SOC to a membrane reactor was successfully realized in this study. The application of this membrane reactor should not be limited to the partial oxidation of methane, and other reactions can be considered by using corresponding membranes.

## Figures and Tables

**Table 1:** Arrhenius parameters of the Arrhenius reaction rate $k_{i}$ of Equations (5)–(8) [15,16].

Arrhenius Reaction Rate k_i_	Pre-Exponential Factor A_i_		Activation Energy E_act,i_	
	Value	Unit	Value	Unit
k_1a_	8.11 × 10^5^	mol/(bar^2^ kg_cat_ s)	86	kJ/mol
k_1b_	6.82 × 10^5^			
k_2_	1.17 × 10^15^	mol bar^0.5^/(kg_cat_ s)	240.1	
k_3_	5.43 × 10^5^	mol/(bar kg_cat_ s)	67.1	
k_4_	2.83 × 10^14^	mol bar^0.5^/(kg_cat_ s)	243.9	

**Table 2:** Arrhenius parameters of adsorption coefficient $K_{j}$ of Equations (5)–(8) [15,16].

Catalyst Adsorption Coefficient K_j_	Pre-Exponential Factor A(K_j_)		Activation Energy ΔH_j_^0^	
	Value	Unit	Value	Unit
K_CH4,ox_	1.26 × 10^−1^	1/bar	−27.3	kJ/mol
K_O2,ox_	7.87 × 10^−7^		−92.8	
K_CH4_	6.65 × 10^−4^		−38.3	
K_CO_	8.23 × 10^−5^		−70.7	
K_H2_	6.12 × 10^−9^		−82.9	
K_H2O_	1.77 × 10^5^	-	88.7	

**Table 3:** Representative oxygen fluxes and operating conditions of potentially applicable ITMs from literature.

Membrane Type	Formula	Temperature (°C)	Feed Oxygen Pressure (bar)	Oxygen Flux (μmol cm^−2^ s^−1^)	Ref.
SCF	SrCo_0.8_Fe_0.2_O_3−δ_	900	0.63	4.5 × 10^−3^	[20,23]
BSCF	Ba_0.5_Sr_0.5_Co_0.8_Fe_0.2_O_3−δ_	700–875	0.21	4 × 10^−1^−1.35	[18]
LSCF	La_0.6_Sr_0.4_Co_0.2_Fe_0.8_O_3−δ_	680–1000	0.21–1.0	3.0 × 10^−4^−1.2	[19]
SCFN	SrCo_x_Fe_0.9-x_Nb_0.1_O_3−δ_	700–900	0.21	9 × 10^−2^−1.5	[22]
LNO	La_2_NiO_4+δ_	850–1000	0.1–1.0	1 × 10^−1^−8 × 10^−1^	[21]
LSGF-BSCF	La_0.1_5Sr_0.85_Ga_0.3_Fe_0.7_O_3−δ_ Ba_0.5_Sr_0.5_Fe_0.2_Co_0.8_Fe_0.2_O_3−δ_	700–915	0.21	1 × 10^−1^−6 × 10^−1^	[18]

**Table 4:** Pre-exponential factor and activation energy of $k_{f}$, $k_{r}$ and $D_{V}$ [19].

Expression	Pre-Exponential Factor		Activation Energy	
	Value	Unit	Value	Unit
$k_{f}=k_{f}^{0}\exp\left(-\frac{E_{f}}{RT}\right)$	(9.21 ± 0.44) × 10^8^	cm^2^/s	268 ± 0.5	kJ/mol
$k_{r}=k_{r}^{0}\exp\left(-\frac{E_{r}}{RT}\right)$	(1.75 ± 0.087) × 10^11^	cm/(atm^0.5^ s)	377 ± 0.4	kJ/mol
$D_{V}=D_{V}^{0}\exp\left(-\frac{E_{D}}{RT}\right)$	(1.01 ± 0.05) × 10^−2^	mol/(cm^2^ s)	75.6 ± 0.5	kJ/mol

**Table 5:** Dimensions of the reconstructed geometry of the fluid domain.

Parameters	Values
Total length	0.18 m
Total width	0.10 m
Diameters of inlets and outlets	0.011 m
Membrane area	4 × 0.003471 m^2^
Membrane thickness	0.001 m
Volume of air domain	2.3168 × 10^−5^ m^3^
Volume of fuel domain	4.3393 × 10^−5^ m^3^

**Table 6:** Settings for the boundary and cell zone.

Boundaries and Zones	Conditions	Settings
Inlets	Inlet type	Velocity inlet
	Velocity	Various, depending on operating conditions
	Temperature	Various, depending on operating conditions
	Turbulence intensity	5%
	Turbulence viscosity ratio	10
	Species mass fractions	Air inlets: 0.23 for O_2_ and 0.77 for N_2_
		Fuel inlets: 1.00 for CH_4_
Outlets	Outlet type	Pressure outlet
Wall of fuel zone	Thermal conditions	Isothermal: constant temperature depends on operating conditions
		Adiabatic: default
Membranes-fuel zone contact surface	Thermal conditions	Isothermal: constant temperature depends on operating conditions
		Adiabatic: coupled
Air zone	Options	Enable source terms
Fuel zone	Options	Enable source terms and reaction

**Table 7:** Product composition, methane conversion rate, and CO selectivity under adiabatic operation ($m_{air}=2.772\times 10^{-5}\;\mathrm{kg}/s$, $m_{fuel}=1.850\times 10^{-7}\;\mathrm{kg}/s$, $T_{air,\;in}=T_{fuel,\;in}=1050\;K$).

Parameters		Value
Species mole fraction in the product	CO	20.68%
	H_2_	38.60%
	CO_2_	12.91%
	H_2_O	27.82%
Methane conversion rate		~100%
CO selectivity		61.60%

**Table 8:** Comparison of reactor performance under isothermal and adiabatic conditions.

	Isothermal Operation	Adiabatic Operation
Operating temperature	1100 K	1050–1244 K
External temperature control	Need	No need
Methane flow rate	7.676 × 10^−7^ kg/s	1.850 × 10^−7^ kg/s
Airflow rate	1.971 × 10^−5^ kg/s	2.772 × 10^−5^ kg/s
Oxygen permeation rate	1.72 × 10^−1^ μmol/(cm^2^s)	9.07 × 10^−2^ μmol/(cm^2^s)
Methane conversion rate	~96%	~100%
CO selectivity	~97%	~62%

## Data Availability

The data presented in this study can be requested from the corresponding author for a reasonable reason.
